# Association of Overnight Fasting Duration and Meal Frequency with Glucose and Lipid Metabolism During Pregnancy: A Cross-Sectional Study

**DOI:** 10.3390/nu17050807

**Published:** 2025-02-26

**Authors:** Keiko Nakano, Moeko Tanaka, Nao Nishihara, Yuriko Usui, Kaori Yonezawa, Naoko Hikita, Emi Tahara-Sasagawa, Satoshi Sasaki, Takeshi Nagamatsu, Megumi Haruna, Tokyo Area Members of the J-PEACH Study Group as of 2019–2022

**Affiliations:** 1 Department of Midwifery and Women’s Health, Division of Health Sciences and Nursing, Graduate School of Medicine, The University of Tokyo, 7-3-1 Hongo, Bunkyo-ku, Tokyo 113-0033, Japan; nakano-keiko144@g.ecc.u-tokyo.ac.jp (K.N.); tanaka-moeko48526@g.ecc.u-tokyo.ac.jp (M.T.); nishihara-nao185@g.ecc.u-tokyo.ac.jp (N.N.); yusui@g.ecc.u-tokyo.ac.jp (Y.U.); kaoriyone@m.u-tokyo.ac.jp (K.Y.); nhikita-tky@umin.ac.jp (N.H.); e-sasagawa@redcross.ac.jp (E.T.-S.); 2 Department of Social and Preventive Epidemiology, School of Public Health, The University of Tokyo, 7-3-1 Hongo, Bunkyo-ku, Tokyo 113-0033, Japan; stssasak@m.u-tokyo.ac.jp; 3 Department of Obstetrics and Gynecology, Graduate School of Medicine, The University of Tokyo, 7-3-1 Hongo, Bunkyo-ku, Tokyo 113-0033, Japan; tnag.tky@gmail.com

**Keywords:** fasting, feeding behavior, glycated serum albumin, meal frequency, pregnancy

## Abstract

**Background/Objectives**: Glucose and lipid metabolism during pregnancy are crucial for perinatal outcomes. Recently, chrono-nutritional factors have been partially identified as influencing pregnancy metabolism. This study aimed to investigate overnight fasting duration and meal frequency during pregnancy and to clarify their associations with glucose and lipid metabolism. **Methods**: This cross-sectional study was conducted at a university hospital in Tokyo, Japan, between February 2020 and June 2021. A self-administered questionnaire was used to evaluate overnight fasting duration and meal frequency in 144 pregnant women in their second trimester. Nutrient intake was assessed using the brief-type self-administered diet history questionnaire. Non-fasting blood samples were collected and analyzed for levels of total cholesterol, triglycerides, high-density lipoprotein cholesterol, low-density lipoprotein cholesterol, and glycated albumin. **Results**: The mean ± standard deviation of overnight fasting duration was 12.1 ± 1.5 h, meal frequency was 3.8 ± 0.9 times per day, and glycated albumin level was 13.3 ± 1.0%. Multivariate analysis revealed that a longer overnight fasting duration was significantly associated with lower glycated albumin levels (β = −0.167, *p* = 0.030). **Conclusions**: These findings suggest that, in addition to meal content and quantity, overnight fasting may be effective in appropriately managing glycated albumin levels during the second trimester of pregnancy.

## 1. Introduction

Glucose and lipid metabolism change significantly during pregnancy. Gestational hyperglycemia has been associated with perinatal outcomes such as fetal macrosomia, neonatal hypoglycemia, neonatal adiposity, and increased risk of childhood obesity. These outcomes are present even when blood glucose levels are below the standard diagnostic values for gestational diabetes mellitus (GDM) [1,2]. Maintaining an adequate level of blood glucose can improve perinatal outcomes [3,4].

Dyslipidemia during pregnancy is associated with GDM, preeclampsia, premature birth, and other adverse outcomes [5,6]. Elevated high-density lipoprotein cholesterol (HDL-C) levels during pregnancy reduce the risk of GDM and macrosomia, while increased triglycerides (TG) during the third trimester increase the risk of GDM, preeclampsia, large for gestational age (LGA), macrosomia, and premature birth [5]. Furthermore, TG levels during early pregnancy are linearly associated with an increased risk of pregnancy-induced hypertension (PIH), preeclampsia, LGA, and premature birth [7]. Low-density lipoprotein cholesterol (LDL-C) in the second trimester is correlated with a higher risk of macrosomia [6]. Therefore, appropriate control of glucose and lipid metabolism during pregnancy is crucial for preventing adverse perinatal outcomes.

Recently, chrono-nutrition has become noticeable. Circadian rhythms are synchronized by light-induced and light-dark cycles and environmental factors. Furthermore, synchronization by mealtime has been studied extensively as an important factor [8,9,10,11]. Since humans’ circadian rhythm regulates glucose and lipid metabolism [12,13], dietary habits, such as intervals between meals (fasting duration) and meal frequency, may have important effects on metabolism. However, in previous studies, the metabolic effects of fasting duration and meal frequency were inconsistent. A meta-analysis suggested that increased meal frequency may be associated with decreased fat mass, decreased body fat percentage, and increased fat-free mass in adults [14]. Nevertheless, a review suggested that a lower meal frequency (without skipping breakfast) with regular fasting times may have physiological benefits such as reduced inflammation, improved circadian rhythm, increased autophagy and stress resistance, and modulation of gut microbiota [15]. These findings indicate that the effects of fasting duration and meal frequency on metabolism remain controversial and require further investigation.

Most studies have focused on diet amount and content to improve glucose and lipid metabolism during pregnancy [16]. Only a few studies have focused on the impact of chrono-nutritional variables such as fasting duration and meal frequency, which should also be considered in pregnant women. A cross-sectional cohort study in Singapore reported that increased overnight fasting duration and decreased meal frequency reduced fasting blood glucose and 2 h oral glucose tolerance test (OGTT) glucose levels, respectively [17]. To assess the effects of overnight fasting duration and meal frequency on glucose metabolism accurately, it is essential to conduct studies over several weeks using biomarkers that remain stable regardless of blood glucose fluctuations (e.g., those caused by recent meals or physical activity) and are not influenced by circadian rhythms. Additionally, there is a lack of previous research examining the impact of these factors on lipid metabolism. Considering that changes in both glucose and lipid metabolism significantly influence the health outcomes of pregnant women and their offspring, further investigation into these effects is warranted.

This study aimed to identify the overnight fasting duration and meal frequency during pregnancy and clarify their effects on glucose and lipid metabolism. This study hypothesized that nutrition intake along the circadian rhythm can be achieved in pregnant women with a long overnight fasting period, and that glucose and lipid metabolism could be successfully maintained. Moreover, this study considered that a higher meal frequency would correspond to a shorter meal interval, and a higher energy intake would result in a higher glucose and lipid metabolism index.

## 2. Materials and Methods

### 2.1. Study Design and Participants

This study is a part of the Japan Pregnancy Eating and Activity Cohort (J-PEACH) study, which was initiated in 2020. The J-PEACH study is a multicenter study (Yamagata, Tokyo, Osaka, and Fukuoka) of lifestyle and health during and after pregnancy [18]. In this study, participants were limited to those from Tokyo because blood samples could only be obtained during antenatal check-ups at the hospital in the city.

This cross-sectional study was conducted at the obstetric outpatient department of a university hospital in Tokyo, Japan, which performs approximately 1000 labors per year. Participants were recruited from February 2020 to June 2021. Due to the novel coronavirus disease 2019 (COVID-19) pandemic, recruitments at the outpatient department were suspended between 2 April and 6 July. The eligibility criteria included pregnant women above the age of 18 years in the first to second trimester, who visited the hospital for antenatal check-ups and could read and write in Japanese. The exclusion criteria were defined as pregnant women who were not scheduled for delivery at the study hospital or were judged by the medical staff as not suitable for participation in the research. Data from women with a history of dyslipidemia or glucose intolerance, those diagnosed with gestational diabetes mellitus (GDM) at the time of questionnaire response or blood sampling, those experiencing severe nausea and vomiting at the time of questionnaire response, and those with multiple pregnancies were excluded from the data analysis to eliminate potential confounding factors.

The required sample size was determined using G*Power 3.1 [19]. The required sample size for performing multiple regression analysis was calculated to be 92 participants, assuming an effect size of 0.15 and five covariates. For comparisons among groups, sample size calculations were based on TG and GA values from previous studies [7,20]. Specifically, a one-way analysis of variance (ANOVA) was used, assuming an effect size of 0.30, an alpha level of 0.05, 80% power, and three groups. This resulted in a final required sample size of 111 women for this study.

### 2.2. Procedures

The eligible participants were recruited by researchers in the outpatient waiting room. Moreover, an e-mail address was obtained from the agreed participants. The self-administered questionnaire survey and blood tests were performed in the second trimester of pregnancy. The questionnaire was initially prepared on paper and filled out while waiting for antenatal check-ups but was switched to an online version in May 2020 due to the COVID-19-triggered suspension of in-hospital surveys. The online questionnaires were e-mailed to participants after gestational week 18, and their responses were received by gestational week 26. The questionnaire was resent only once to participants who did not respond within 2 weeks of the initial e-mail.

### 2.3. Data Collection and Measurements

#### 2.3.1. Overnight Fasting Duration and Meal Frequency

Meal frequencies were defined as events in which the participant consumed ≥50 kcal, with a time interval between eating episodes of ≥15 min, as described in previous studies [17,21,22]. This definition of meal frequency predicts the total energy intake sufficiently [23]. In this study, the participants answered a questionnaire regarding their habitual eating habits over the previous month. Overnight fasting duration was calculated as the time from the last finished meal (on a typical day) to the first meal of the next day. The meal frequency was estimated based on the number of eating episodes (including light meals and snacks). The two participants who reported zero or one meal were excluded from the analysis using the meal frequency variable, as their responses to other questions indicated they had eaten at least twice, suggesting invalid responses.

#### 2.3.2. Biomarker Analyses

Non-fasting blood samples were collected as part of antenatal check-up blood draws from participants in the second trimester of pregnancy (17–22 gestational weeks). All serum samples were assayed for total cholesterol (TC), TG, HDL-C, LDL-C, and glycated albumin (GA) concentrations. The biomarkers were measured by a contract laboratory (SRL, Inc., Tokyo, Japan). TC, TG, and GA were analyzed by enzymatic methods, and HDL-C and LDL-C were analyzed by direct methods, all using an Ultra High Throughput Clinical Chemistry Analyzer, JCA-BM8000 series (JEOL Ltd., Tokyo, Japan).

Fasting blood glucose and glycosylated hemoglobin (HbA1c), which are used as diagnostic criteria for GDM, are the indicators of glucose metabolism during pregnancy. However, in this study, GA was measured because, unlike HbA1c, GA is not affected by iron deficiency anemia [24], and GA reflects 2–4 weeks of glucose status (compared to 1–2 months of HbA1c screening), indicating the ability to measure short-term glycemic changes [20]. GA is also considered a useful predictor of neonatal outcomes in pregnant women with GDM [25,26].

#### 2.3.3. Dietary Assessment

Dietary intake was assessed using a brief-type self-administered diet history questionnaire (BDHQ) [27,28] which was validated for Japanese pregnant women [29]. The BDHQ is a questionnaire designed to examine habitual nutrient intake and obtain information on individual nutrient intake, food intake, and other qualitative food behavioral indicators. The BDHQ consists of questions regarding eating habits over the previous month and requires 15 min to complete. The estimates of the dietary intakes of 58 foods and beverages, along with energy and selected nutrients, were calculated by an ad hoc computer algorithm (including weighting factors for BDHQ). In the analyses, food group and nutrient intakes were energy-adjusted using the density method to reduce intra-individual measurement errors. Participants with unrealistic energy intake were excluded from the analysis. The exclusion criteria included an energy intake value of less than 0.5 and an energy requirement of more than 1.5 times compared to the average for pregnant women who were moderately physically active [30].

#### 2.3.4. Other Measurements

Demographic characteristics: Data on age, parity, medical history, and the expected date of delivery were collected from medical records. The gestational age at the time of questionnaire response and at the time of blood sampling was calculated based on the respective dates and the expected delivery date. Moreover, marital status, educational status, annual household income, working status, night shift, bedtime, and sleep duration information were collected from questionnaires. In the questionnaire, time was estimated using the 24-hour system, but the values were revised accordingly for cases in which time was estimated using the 12-hour system. Moreover, sleep duration was calculated using wake-up time and bedtime.

Pre-pregnancy body mass index (BMI): Pre-pregnancy weight and height were collected from medical records or questionnaire data in case of unavailability of the former. BMI was calculated by dividing weight (kg) by height (m^2^). BMI was classified into three groups: underweight (<18.5 kg/m^2^), normal weight (18.5–24.9 kg/m^2^), and overweight (≥25.0 kg/m^2^) groups on the World Health Organization BMI classification [31].

Physical symptoms and activity: Nausea and vomiting were evaluated using a modified Pregnancy-Unique Quantification of Emesis and Nausea (m-PUQE) for symptoms over the past 1 month [32,33]. Participants were divided into two groups according to m-PUQE total scores: (a) mild (≤6) and (b) moderate (7–12). Women classified as having severe symptoms (≥13) were excluded from the analysis to account for the potential impact on eating behavior.

Physical activity was evaluated using the Pregnancy Physical Activity Questionnaire translated into Japanese 2020 (PPAQ-J 2020) with some modifications of the validated PPAQ-J [34,35]. The activity levels classified as moderate and vigorous, were summed, and then classified into two groups: the inactive group with a total activity level of <7.5 metabolic equivalents of task (METs)-hours/week and the active group with a total activity level of ≥7.5 METs-hours/week [36].

### 2.4. Statistical Analysis

Categorical data were presented as frequencies and percentages, while normally distributed continuous data and non-normally distributed continuous data were, respectively, presented as means ± standard deviation (SD) and median (interquartile range, IQR). Normality was checked using a histogram, boxplot, q-norm plot, kurtosis, and skewness. In terms of overnight fasting duration, individuals were grouped into tertiles. With respect to meal frequency, the groups were 2–3, 4, and 5–7 times/day. ANOVA was used to compare the averages of normally distributed continuous variables, the Kruskal–Wallis test to compare non-normally continuous variables, and Pearson’s chi-square test and Fisher’s exact test to compare the proportions of categorical variables between the groups. Pearson’s correlation coefficients were calculated to examine the associations between GA, overnight fasting duration, meal frequency, BMI, and age, as well as the associations between overnight fasting duration, meal frequency, and lipid metabolism parameters. Multiple linear regressions were performed to examine the associations of overnight fasting duration and meal frequency with GA. Duration of overnight fasting and meal frequency were included as continuous variables in the same model and adjusted for covariates. The covariates used for adjustment were BMI and age, as identified in previous studies [37,38]. Any missing data were excluded from analyses, while *p* < 0.05 was considered statistically significant. All the statistical analyses were performed using SPSS version 29.0 for Microsoft Windows (IBM Corp., Armonk, NY, USA).

## 3. Results

### 3.1. Participants’ Characteristics

**Figure 1:**
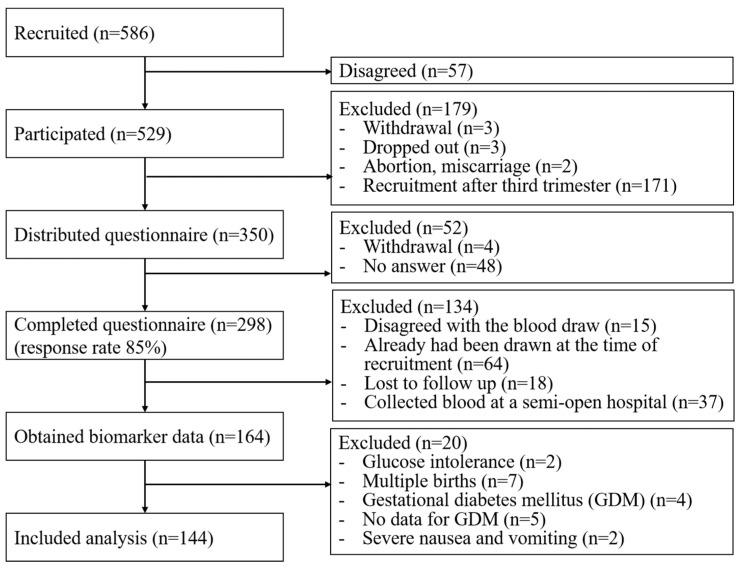
Flowchart of the study participants.

In the present study, 586 women were recruited for the J-PEACH study and 529 (90%) women consented to participate in the study. Consent was not obtained from 57 women. The questionnaire was distributed to 350 women between 18 and 24 weeks of gestation. Among them, 293 women completed the questionnaire online, while five of them provided hard copies. Hence, the overall response rate was 85%. Biomarker data were obtained from 164 respondents, and 20 of them were excluded owing to a history of glucose intolerance in two participants, multiple pregnancies in six participants, GDM in five participants, no data available to diagnose GDM in two participants, and severe nausea and vomiting in two participants. Finally, 144 pregnant women were included in this study (Figure 1). There were no significant differences in any characteristics between the populations with and without biomarker data.

Table 1 describes the participants’ characteristics. The mean ± SD time of overnight fasting duration was 12.1 ± 1.5 h, and the mean ± SD number of meal frequency was 3.8 ± 0.9 times/day. Women with a longer overnight fasting duration were more likely not to be working (*p* = 0.002) and physically inactive (*p* = 0.012) and had a longer sleep duration (*p* < 0.001). No significant differences in any characteristics were observed among the three meal frequency groups.

### 3.2. Associations of Overnight Fasting Duration and Meal Frequency on Lipid and Glucose Metabolism

The data presented in Table 2 are the mean ± SD of metabolic biomarkers for all participants, categorized by three groups of overnight fasting duration and meal frequency. No significant differences were observed among the groups in lipid parameters (TC, TG, HDL-C, and LDL-C) or in the glucose parameter GA.

The Pearson’s correlations between GA levels, overnight fasting duration, meal frequency, pre-pregnancy BMI, and maternal age are shown in Table 3. Meal frequency was positively correlated with GA levels (*r* = 0.196, *p* = 0.010). In contrast, overnight fasting duration and pre-pregnancy BMI were inversely correlated with GA levels (*r* = −0.185, *p* = 0.014, and *r* = −0.450, *p* < 0.001, respectively). No correlations were found among overnight fasting duration, meal frequency, and lipid metabolism parameters.

The association between overnight fasting duration, meal frequency, and GA levels is shown in Table 4. Univariate regression analysis revealed that overnight fasting duration and meal frequency were significantly associated with GA levels. However, in multivariate regression analysis adjusted for BMI and age, only overnight fasting duration showed a significant association with GA levels (β = −0.167, *p* = 0.030).

### 3.3. Association Between Overnight Fasting Duration, Meal Frequency, and Dietary Intake

Table 5 presents the differences in energy, nutrient, and food intake values according to overnight fasting duration and meal frequency. No significant differences were observed in energy or macronutrient intake between the groups. Significant differences were noted in the intake of oils and fats by overnight fasting duration (*p* = 0.016), fruits by overnight fasting duration (*p* = 0.025), and soy products by meal frequency (*p* = 0.032).

## 4. Discussion

To the best of our knowledge, this study is one of the first to investigate the effects of overnight fasting duration and meal frequency during pregnancy on glucose and lipid metabolism. The results indicated that women in the second trimester had a mean overnight fasting duration of 12.1 h and a mean meal frequency of 3.8 meals per day. Furthermore, longer overnight fasting duration was associated with lower GA levels. In contrast, differences in meal frequency did not influence GA levels, and neither overnight fasting duration nor meal frequency affected lipid metabolism. Additionally, no significant differences in energy or macronutrient intake were observed across groups stratified by overnight fasting duration or meal frequency. However, significant differences were identified in the intakes of oils and fats, fruits, and soy products, whereas most other food groups showed no differences.

### 4.1. Characteristics of Study Participants Compared with Other Japanese Cohorts

In this study, glucose and lipid metabolism markers in serum were investigated during the second trimester of pregnancy (17–22 gestational weeks). In a large cohort study in Japan that used non-fasting blood tests similar to this study, the results of median values (IQR) of TC, TG, HDL-C, and LDL-C at 20–23 weeks of gestation were 211 (189–235), 133 (103–171), 79 (70–89), and 115 (97–135) mg/dL, respectively [39]. The values observed in this study were within the indicative ranges. In another Japanese study, the mean ± SD of the GA level in 196 women in the second trimester (14–27 weeks of gestation) was 13.7 ± 1.9% [20], which was higher than the value of 13.3 ± 1.0% observed in this study. Thus, GA levels in this study were lower than those reported in the second-trimester population in Japan. However, the previous study excluded women with a BMI < 18.5 or ≥25, whereas the present study included them. Given that higher BMI is associated with associated GA levels, this difference should be taken into account when comparing results. Compared with the National Health and Nutrition Examination Survey (30–39 years old) and the large cohort study in Japan [40,41], the proportions of pre-pregnancy BMI in this study were lower for participants classified as underweight and obese, and higher for those classified as having normal weight. Moreover, participants in this study were older, more highly educated, and had higher incomes compared to the previous study [40]. Therefore, they might have had prior knowledge regarding diet and sleep, as well as a high level of health consciousness.

### 4.2. Overnight Fasting Duration and Meal Frequency in Pregnant Japanese Women 

This study is the first in Japan in which the overnight fasting duration of pregnant women has been documented; therefore, direct comparisons with the typical overnight fasting duration in Japan are not feasible. However, sleep duration was positively associated with overnight fasting duration and could be compared with national averages. According to a national survey, the mean sleep duration for women aged 35–39 years in Japan is 7.5 h [42]. Similarly, based on a large cohort study in Japan, it was reported that 7–8 h was the most common sleep duration both before and during pregnancy [43]. In our study population, the average sleep duration was 8.0 h, reflecting the lifestyle patterns of the general Japanese population. In contrast, meal frequency among pregnant Japanese women in this study tended to be lower than that reported in two studies from other countries in which the same definition of meal frequency applies. In Singapore, the average meal frequency during 26–28 weeks of gestation is 4.2 times [17], whereas in Brazil, it is 4.7 times during the first trimester [22].

### 4.3. Effect of Overnight Fasting Duration on GA

The GA values of all the participants were within the normal glycemic reference range (15.7% or less) defined by the Japan Diabetes Society [44]. However, even when GA values are within the acceptable range, variations in these values may affect perinatal outcomes. In a previous study, it was suggested that a GA value below the GDM threshold could increase the risk of adverse perinatal outcomes, such as respiratory disorders and large-for-date status [45]. Thus, even within the reference range, a further reduction in GA levels may be clinically relevant for minimizing the risk of such complications. In this study, a longer overnight fasting duration was associated with lower GA values, suggesting a potential benefit. This finding is consistent with previous studies examining the relationship between overnight fasting duration and glucose metabolism indicators during pregnancy, which reported that a longer overnight fasting duration was linked to lower fasting glucose levels [17]. In the present study, which focused on GA, a similar trend was observed, indicating that a longer overnight fasting duration was associated with lower glucose metabolism indicators. Analysis based on the tertiles of overnight fasting duration revealed no impact on meal frequency or energy intake. This suggests that the longer overnight fasting duration was not due to skipping meals, nor did it result in a reduction in energy intake. In experimental studies using mice, time-restricted eating (TRE) has been shown to improve glucose tolerance by reducing insulin resistance [12,46]. Similarly, a longer overnight fasting duration, which results in a more restricted eating window, may have contributed to improved glucose tolerance, leading to lower GA levels. Since glucose metabolism undergoes significant changes during pregnancy, including increased insulin resistance and enhanced insulin secretion to support fetal growth and development [47,48], the influence of overnight fasting duration may be even more significant during pregnancy compared to the non-pregnant state.

It is important to emphasize that a longer overnight fasting duration is not necessarily beneficial. Previous studies have reported no serious adverse effects when pregnant women in the second trimester practiced 10 h of TRE, equivalent to a 14 h overnight fast, for five weeks [49]. Conversely, prolonged fasting durations of 14–18 h, such as skipping breakfast during pregnancy, have been reported to potentially lead to accelerated starvation [50]. These findings suggest that excessively long fasting periods may be harmful.

No significant effect of meal frequency on GA was observed. However, this outcome may be attributed to the fact that the participants in this study reported fewer meals than those in a previous study [17]. Additionally, there were few participants with a meal frequency of 5–10 meals, a range that was associated with changes in glucose metabolism in a previous study [17].

### 4.4. Effects of Meal Frequency and Overnight Fasting Duration on Lipid Metabolism

The results of this study suggest that overnight fasting and meal frequency do not affect lipid metabolism. To date, no other study has investigated the relationship between eating behaviors and lipid metabolism parameters in pregnant women. Several studies have examined TRE and lipid metabolism in adults. However, review articles have concluded that the associations between triglycerides, cholesterol subtypes, and TRE are inconsistent [51,52]. Further research is required to explore the effects of overnight fasting and meal frequency on lipid metabolism during pregnancy.

### 4.5. Effects of Overnight Fasting Duration and Meal Frequency on Dietary Intake

Dietary habits investigated using BDHQ had similar results to a Singapore study that also found no differences in the energy intake of macronutrients by overnight fasting duration or meal frequency [17]. Although a study from Singapore and a study from Brazil revealed that daily energy intake decreased with longer overnight fasting and increased with more frequent meals [17,22], the present study had a different trend in energy intake. In our study population, participants received the necessary nutrients even if the fasting time had been increased, and the total energy intake was suppressed even if the meal frequency had been increased. Furthermore, the group with longer overnight fasting duration tended to have lower GA values; however, there were no observed changes in dietary content, such as avoidance of confectionery and fruits. Additionally, oils, fats, and fruits, which showed significant differences among the tertile groups, did not demonstrate a tendency to explain the decrease in GA values.

### 4.6. Limitations and Strengths

This study has several limitations. First, as a cross-sectional study, causality is not clear. However, this study is part of a cohort study with further ongoing longitudinal studies being conducted to determine causality. Second, there might have been reporting errors, specifically since overnight fasting durations and meal frequencies are based on self-reported results. However, this bias was mitigated by measures, such as providing 50 kcal food samples in the questionnaire. Moreover, although a validated dietary assessment was used, the estimates of energy intake reporting errors [53] require careful interpretation. To reduce this bias for nutrients and food groups, adjustments were made with estimated declaration errors and comparisons were made using density methods. In this way, the bias was minimized. Third, the study population consisted of highly educated, high-income, pregnant women in a metropolitan hospital. Nutrient and food group intake may differ depending on household income, and the content of dietary intake may differ from that of the general population [41]. Furthermore, it is possible that there are many people who pay attention to health and nutrition because they are highly educated on the subject. Additionally, COVID-19 might have affected the participants’ lifestyle by increasing the time spent at home and decreasing the time spent commuting, which may have affected the time for sleeping and eating. This effect should be tested in the cohort when the COVID-19 effects are no longer applicable.

Despite these limitations, this is the first study to examine the actual conditions of overnight fasting duration and meal frequency during pregnancy and their effects on glucose and lipid metabolism in Japan. The strength of the study is that the effect of eating time on biomarker index was quantitatively examined. Furthermore, the analysis of the meal quality, as well as the timing of meals by the BDHQ, was also considered as an important point. Therefore, further studies regarding their effects on perinatal outcomes are required.

## 5. Conclusions

This study is the first to clarify the status of overnight fasting duration and meal frequency among pregnant women in Japan. The findings suggest that overnight fasting duration during the second trimester may influence GA values. This suggests that fasting duration could play a crucial role in enhancing glucose metabolism and potentially reducing the risk of adverse outcomes, even when GA values remain within the normal range. To optimize glycemic control in the second trimester, providing appropriate guidance on managing overnight fasting duration, alongside meal quantity and composition, may be beneficial. Further research is warranted to elucidate the underlying mechanisms and clinical significance of these findings in pregnant populations, particularly in relation to neonatal health outcomes.

## Figures and Tables

**Table 1:** Participant Characteristics Stratified by Overnight Fasting Duration and Meal Frequency.

	All	Overnight Fasting Duration					Meal Frequency				
Variables	(n = 144)	Tertile 1 9.00–11.49 h (n = 48)	Tertile 2 11.50–12.49 h (n = 48)	Tertile 3 12.50–17.00 h (n = 48)	*p*		Group 1 2–3 Times/Day (n = 56)	Group 2 4 Times/Day (n = 53)	Group 3 5–7 Times/Day (n = 33)	*p*	
Age, years	35.3 ± 4.3	35.8 ± 4.4	36.1 ± 4.2	34.2 ± 4.2	0.079	^a^	35.4 ± 4.6	35.3 ± 4.3	35.3 ± 3.7	0.993	^a^
Gestational age, weeks	19.3 ± 1.1	19.1 ± 1.2	19.4 ± 1.2	19.4 ± 1.2	0.348	^a^	19.3 ± 1.1	19.3 ± 1.1	19.2 ± 13	0.847	^a^
Married	141 (97.9)	47 (97.9)	47 (97.9)	47 (97.9)	1.000	^c^	55 (98.2)	51 (96.2)	33 (100.0)	0.616	^c^
Primipara	95 (66.0)	35 (72.9)	25 (52.1)	35(72.9)	0.048	*^,b^	38 (67.9)	35 (66.0)	22 (66.7)	1.000	^b^
Pre-pregnancy BMI, kg/m^2^											
<18.5	21 (14.6)	8 (16.7)	5 (10.4)	8 (16.7)	0.899	^b^	10 (17.9)	4 (7.5)	7 (21.2)	0.208	^c^
≥18.5, and <25	105 (72.9)	35 (72.9)	36 (75.0)	34 (70.8)			37 (66.1)	43 (83.1)	24 (72.7)		
≥25	18 (12.5)	5 (10.4)	7 (14.6)	6 (12.5)			9 (16.1)	6 (11.3)	2 (6.1)		
Education											
Junior High School/High school	5 (3.5)	2 (4.2)	1 (2.1)	2 (4.2)	0.268	^c^	1 (1.8)	3 (5.7)	1 (3.0)	0.355	^c^
Vocational School/Junior College	26 (18.1)	8 (16.7)	10 (20.8)	8 (16.7)			7 (12.5)	11 (20.8)	8 (24.2)		
University	81 (56.3)	22 (45.8)	27 (56.3)	32 (66.7)			37 (66.1)	28 (52.8)	14 (42.4)		
Graduate School	32 (22.2)	16 (33.3)	10 (20.8)	6 (12.5)			11 (19.6)	11 (20.8)	10 (30.3)		
Annual household income											
<7 million Japanese yen	37 (25.7)	13 (27.1)	12 (25.0)	12 (25.0)	1.000	^b^	11 (19.6)	17 (32.1)	8 (24.2)	0.353	^b^
≥7 million Japanese yen	107 (74.3)	35 (72.9)	36 (75.0)	36 (75.0)			45 (80.4)	36 (67.9)	25 (75.8)		
Work (Yes)	103 (71.5)	41 (85.4)	36 (75.0)	26 (54.2)	0.002	*^,b^	40 (71.4)	38 (71.7)	25 (75.8)	0.915	^b^
Night shift (Yes)	1 (0.7)	1 (2.1)	0 (0.0)	0 (0.0)	1.000	^c^	1 (1.8)	0 (0.0)	0 (0.0)	1.000	^c^
Nausea and vomiting (modified PUQE)											
Mild	115 (79.9)	40 (83.3)	41 (85.4)	34 (70.8)	0.183	^b^	42 (75.0)	46 (86.8)	25 (75.8)	0.255	^b^
Moderate	29 (20.1)	8 (16.7)	7 (14.6)	14 (29.2)			14 (25.0)	7 (13.2)	8 (24.2)		
Physical activity (PPAQ-J 2020)											
Inactive (<7.5METs-hours/week)	77 (53.5)	23 (47.9)	20 (41.7)	34 (70.8)	0.012	*^,b^	34 (60.7)	28 (52.8)	15 (45.5)	0.371	^b^
Active (≥7.5METs-hours/week)	67 (46.5)	25 (52.1)	28 (58.3)	14 (29.2)			22 (39.3)	25 (47.2)	18 (54.5)		
Sleep duration, hours	8.0 ± 1.2	7.1 ± 0.9	8.0 ± 0.9	8.8 ± 1.1	<0.001	*^,a^	8.0 ± 1.2	7.9 ± 1.1	8.0 ± 1.2	0.917	^a^
Bedtime, 24 h system	23:25 ±1:13	23:35 ± 0:59	23:00 ± 0:54	23:40 ± 1:33	0.013	*^,a^	23:28 ± 1:16	23:35 ± 1:12	23:07 ± 1:02	0.213	^a^
Overnight fasting duration, hours	12.1 ± 1.5	10.6 ± 0.7	11.9 ± 0.3	13.7 ± 1.2	<0.001	*^,a^	12.2 ± 1.7	11.8 ± 1.5	12.1 ± 1.2	0.366	^a^
Meal Frequency, per day (n = 142)	3.8 ± 0.9	3.9 ± 1.0	3.9 ± 0.9	3.7 ± 1.0	0.519	^a^	2.9 ± 0.3	4.0 ± 0.0	5.2 ± 0.5	<0.001	*^,a^

Values are means ± SDs or n (%). SD, standard deviation; BMI, body mass index; PUQE, Pregnancy-Unique Quantification of Emesis Scoring Index; PPAQ-J 2020, Pregnancy Physical Activity Questionnaire translated in Japan 2020; MET, metabolic equivalents of task. Participants were categorized into tertiles based on overnight fasting duration (Tertile 1, Tertile 2, Tertile 3) and into three groups based on meal frequency (Group 1: 2–3 times/day, Group 2: 4 times/day, Group 3: 5–7 times/day). * *p* < 0.05 was considered statistically significant. ^a^ one-way analysis of variance, ^b^ Pearson’s chi-square test, ^c^ Fisher’s exact test.

**Table 2:** Values of lipid and glucose parameters in the total population and stratified by overnight fasting duration and meal frequency.

	All	Overnight Fasting Duration				Meal Frequency			
Variables	(n = 144)	Tertile 1 9.00–11.49 h (n = 48)	Tertile 2 11.50–12.49 h (n = 48)	Tertile 3 12.50–17.00 h (n = 48)	*p*	Group 1 2–3 Times/Day (n = 56)	Group 2 4 Times/Day (n = 53)	Group 3 5–7 Times/Day (n = 33)	*p*
TC, mg/dL	228.6 ± 33.2	222.8 ± 30.4	235.3 ± 36.4	227.6 ± 32.2	0.178	233.9 ± 36.5	228.2 ± 32.5	219.2 ± 27.7	0.132
TG, mg/dL	162.5 ± 63.0	156.6 ± 70.5	165.9 ± 59.0	165.1 ± 59.8	0.729	170.9 ± 68.4	150.1 ± 55.2	166.8 ± 64.0	0.202
HDL-C, mg/dL	81.9 ± 13.5	80.8 ± 12.9	80.9 ± 12.9	84.2 ± 14.5	0.375	81.5 ± 13.5	84.9 ± 14.0	77.9 ± 11.7	0.064
LDL-C, mg/dL	123.2 ± 27.8	120.1 ± 26.4	129.8 ± 29.1	119.6 ± 27.1	0.127	128.3 ± 29.4	120.7 ± 26.9	117.8 ± 25.3	0.172
GA, %	13.3 ± 1.0	13.5 ± 1.1	13.3 ± 0.9	13.1 ± 0.9	0.128	13.1 ± 0.9	13.3 ± 1.0	13.6 ± 1.1	0.076

Values are means ± SDs. One-way analysis of variance was performed. SD, standard deviation; TC, total cholesterol; TG, triglycerides; HDL-C, high-density lipoprotein cholesterol; LDL-C, low-density lipoprotein cholesterol; GA, glycated albumin. Participants were categorized into tertiles based on overnight fasting duration (Tertile 1, Tertile 2, Tertile 3) and into three groups based on meal frequency (Group 1: 2–3 times/day, Group 2: 4 times/day, Group 3: 5–7 times/day).

**Table 3:** Pearson’s correlation coefficient between GA, overnight fasting duration, meal frequency, BMI, and age.

Variables	1	2	3	4
1. GA				
2. Overnight fasting duration	−0.185 *			
3. Meal frequency	0.196 *	−0.116		
4. Pre-pregnancy BMI	−0.450 **	0.017	−0.104	
5. Maternal age	−0.019	−0.235 *	0.017	0.086

GA, glycated albumin; BMI, body mass index. * *p* < 0.05, ** *p* < 0.001.

**Table 4:** Associations of overnight fasting duration and meal frequency with GA.

	Crude							Model ^a^						
	Unstandardized Coefficients		Standardized Coefficients			95% CI for B		Unstandardized Coefficients		Standardized Coefficients			95% CI for B	
	B	SE	β	t	*p*	LB	UB	B	SE	β	t	*p*	LB	UB
Overnight fasting duration	−0.135	0.054	−0.206	−2.506	0.013 *	−0.241	−0.028	−0.109	0.050	−0.167	−2.188	0.030 *	−0.207	−0.010
Meal frequency	0.210	0.089	0.196	2.366	0.019 *	0.035	0.385	0.142	0.080	0.132	1.768	0.079	−0.017	0.300

Adjusted R^2^ = 0.252, *p* < 0.001. Multiple linear regressions were performed. GA, glycated albumin; CI, confidence intervals; B, unstandardized regression coefficients; SE, standard error; LB, lower bound; UB, upper bound. ^a^ Model was adjusted for pre-pregnancy body mass index and maternal age. * *p* < 0.05 was considered statistically significant.

**Table 5:** Comparison of energy-adjusted nutrient intakes and food group intakes by overnight fasting duration and meal frequency.

	ALL	Overnight Fasting Duration			Meal Frequency				
	(n = 112)	Tertile 1 9.00–11.49 h (n = 37)	Tertile 2 11.50–12.49 h (n = 39)	Tertile 3 12.50–17.00 h (n = 36)	*p*	Group 1 2–3 Times/Day (n = 37)	Group 2 4 Times/Day (n = 45)	Group 3 5–7 Times/Day (n = 29)	*p*
Energy and nutrients									
Energy (kcal/day)	1536 (1333–1803)	1585 (1409–1825)	1515 (1427–1833)	1510 (1268–1650)	0.317	1533 (1356–1798)	1566 (1354–1832)	1544 (1329–1728)	0.891
Protein (% of energy)	14.4 (13.2–16.3)	14.5 (12.9–16.1)	14.6 (13.3–16.5)	14.2 (13.1–16.1)	0.521	14.7 (13.2–16.8)	14.6 (13.3–16.5)	13.9 (12.8–15.3)	0.076
Fat (% of energy)	28.9 (26.1–33.1)	28.9 (26.5–33.2)	28.6 (25.4–32.6)	30.2 (26.1–33.9)	0.807	29.0 (25.3–32.2)	29.6 (26.3–34.2)	28.5 (25.5–33.1)	0.583
Carbohydrate (% of energy)	55.1 (50.9–58.9)	56.1 (48.4–59.2)	55.2 (51.6–58.6)	54.8 (50.5–59.0)	0.996	54.9 (50.7–58.4)	52.7 (47.6–58.7)	56.5 (52.1–60.5)	0.252
Dietary fiber (g/1000 kcal)	6.6 (5.7–7.7)	6.6 (5.7–7.3)	6.6 (5.4–7.8)	6.7 (5.8–7.8)	0.906	6.8 (5.9–8.6)	6.6 (5.8–7.7)	6.2 (5.3–7.1)	0.140
Food group (g/1000 kcal)									
Cereals	221 (175–276)	234 (181–276)	218 (179–282)	221 (159–276)	0.815	236 (180–287)	219 (175–263)	208 (174–279)	0.572
Potatoes	20.7 (11.8–32.6)	24.3 (12.2–31.9)	16.7 (10.9–32.9)	20.1 (11.4–34.2)	0.925	19.1 (11.5–37.5)	23.4 (11.4–32.5)	19.2 (12.5–30.4)	0.943
Soy products	27.3 (13.4–41.2)	20.6 (12.0–33.6)	34.6 (17.5–50.7)	26.2 (10.7–38.9)	0.052	35.0 (17.5–54.6)	28.7 (11.2–42.1)	21.0 (12.4–28.6)	0.032 *
Sugar	1.76 (1.29–2.64)	1.60 (1.25–2.35)	1.87 (1.56–2.83)	1.56 (1.01–2.93)	0.224	1.57 (1.22–2.61)	1.85 (1.27–2.82)	1.74 (1.41–2.59)	0.711
Confectioneries	28.0 (17.3–40.6)	28.3 (17.8–39.5)	26.2 (14.4–39.7)	31.2 (16.4–43.9)	0.907	26.8 (12.8–37.7)	26.9 (17.4–38.1)	34.6 (19.0–48.8)	0.155
Oils and fats	6.5 (5.1–8.7)	7.3 (5.8–9.4)	5.9 (4.7–7.3)	6.8 (4.6–8.4)	0.016 *	6.8 (5.0–8.1)	6.4 (5.3–9.5)	6.5 (5.0–9.1)	0.719
Seasonings	10.7 (8.5–12.7)	11.0 (8.5–12.6)	10.7 (8.8–12.8)	9.4 (7.3–13.2)	0.744	11.1 (9.2–14.1)	10.7 (8.6–12.7)	9.1 (6.5–12.4)	0.053
Fruits	55.2 (38.4–82.5)	62.0 (43.9–84.9)	45.4 (21.1–70.0)	68.3 (45.0–94.6)	0.025 *	46.8 (30.9–75.3)	50.8 (37.1–83.7)	56.6 (39.5–78.7)	0.101
Green and yellow vegetables	59.3 (39.9–84.5)	59.8 (41.2–82.8)	47.1 (36.7–88.3)	65.2 (47.1–88.2)	0.566	69.7 (45.0–86.5)	50.8 (44.8–87.8)	58.4 (38.4–82.0)	0.317
Other vegetables	64.1 (45.2–87.3)	61.4 (44.4–70.6)	58.9 (38.9–97.9)	74.5 (45.6–103.6)	0.246	65.4 (39.3–95.1)	68.0 (50.6–89.9)	54.8 (39.9–74.3)	0.157
Mushrooms	6.1 (3.3–8.2)	5.8 (2.7–8.6)	6.5 (3.4–8.6)	6.1 (3.2–7.6)	0.769	5.8 (2.8–7.6)	6.7 (3.8–9.5)	5.7 (3.4–7.3)	0.280
Seaweeds	2.9 (1.5–7.1)	3.5 (1.6–7.5)	2.9 (1.5–7.5)	2.0 (1.3–4.1)	0.148	2.9 (1.6–7.2)	3.7 (1.4–7.9)	1.7 (1.3–5.1)	0.275
Non-alcoholic beverages	75.8 (20.7–134.3)	56.5 (25.8–127.0)	114.1 (21.2–147.9)	60.2 (13.6–124.0)	0.215	66.8 (14.1–117.9)	78.2 (21.9–136.8)	76.4 (36.3–147.8)	0.338
Fish and shellfish	26.1 (17.8–41.6)	26.2 (18.3–40.9)	27.7 (21.1–49.0)	21.8 (13.2–37.5)	0.059	26.5 (15.5–46.5)	27.5 (19.6–48.4)	21.1 (15.0–29.8)	0.109
Meat	42.1 (33.2–52.0)	41.1 (32.1–54.2)	39.5 (31.8–52.0)	43.4 (35.4–53.1)	0.464	39.7 (29.2–51.3)	43.1 (36.6–58.2)	41.4 (31.5–51.0)	0.345
Eggs	16.2 (11.9–26.9)	17.9 (11.9–30.7)	15.8 (12.3–25.3)	17.5 (9.3–28.5)	0.958	17.7 (12.0–28.2)	15.1 (11.3–22.7)	18.1 (11.7–28.7)	0.606
Daily products	98.6 (63.4–129.9)	96.7 (46.0–150.0)	97.3 (69.1–126.5)	109.3 (64.1–131.8)	0.845	92.3 (55.8–124.3)	102.4 (70.5–132.9)	96.9 (63.1–161.5)	0.590

Values are median (IQR). Kruskal–Wallis test was performed. IQR, interquartile range. Participants were categorized into tertiles based on overnight fasting duration (Tertile 1, Tertile 2, Tertile 3) and into three groups based on meal frequency (Group 1: 2–3 times/day, Group 2: 4 times/day, Group 3: 5–7 times/day). * *p* < 0.05 was considered statistically significant.

## Data Availability

The data presented in this study are not publicly available due to ethical considerations and the protection of personal information. However, these data will be made available upon reasonable request to the corresponding author.

## Appendix A

Members in Tokyo area of the J-PEACH Study as of 2019–2022: Megumi Haruna (Principal Investigator), Satoshi Sasaki, Naoko Hikita, Emi Sasagawa, Kaori Yonezawa, Yuriko Usui, Keiko Nakano, Moeko Tanaka, Nao Nishihara, Riko Ohori, Nana Wakabayashi, Satoko Aoyama, and Tomomi Ohyama (The University of Tokyo, Japan).
